# Granzyme B mediated function of Parvovirus B19-specific CD4^+^ T cells

**DOI:** 10.1038/cti.2015.13

**Published:** 2015-07-03

**Authors:** Arun Kumar, Maria F Perdomo, Anu Kantele, Lea Hedman, Klaus Hedman, Rauli Franssila

**Affiliations:** 1 Department of Virology, Haartman Institute, University of Helsinki, Helsinki, Finland; 2 Department of Bacteriology and Immunology, Haartman Institute, University of Helsinki, Helsinki, Finland; 3 Division of Infectious Diseases, Helsinki University Central Hospital, Helsinki, Finland; 4 Department of Medicine, Institute of Clinical Medicine, University of Helsinki, Helsinki, Finland; 5 Helsinki University Central Hospital Laboratory Division, Department of Virology and Immunology, Helsinki, Finland

## Abstract

A novel conception of CD4^+^ T cells with cytolytic potential (CD4^+^ CTL) is emerging. These cells appear to have a part in controlling malignancies and chronic infections. Human parvovirus B19 can cause a persistent infection, yet no data exist on the presence of B19-specific CD4^+^ CTLs. Such cells could have a role in the pathogenesis of some autoimmune disorders reported to be associated with B19. We explored the cytolytic potential of human parvovirus B19-specific T cells by stimulating peripheral blood mononuclear cell (PBMC) with recombinant B19-VP2 virus-like particles. The cytolytic potential was determined by enzyme immunoassay-based quantitation of granzyme B (GrB) and perforin from the tissue culture supernatants, by intracellular cytokine staining (ICS) and by detecting direct cytotoxicity. GrB and perforin responses with the B19 antigen were readily detectable in B19-seropositive individuals. T-cell depletion, HLA blocking and ICS experiments showed GrB and perforin to be secreted by CD4^+^ T cells. CD4^+^ T cells with strong GrB responses were found to exhibit direct cytotoxicity. As anticipated, ICS of B19-specific CD4^+^ T cells showed expected co-expression of GrB, perforin and interferon gamma (IFN-γ). Unexpectedly, also a strong co-expression of GrB and interleukin 17 (IL-17) was detected. These cells expressed natural killer (NK) cell surface marker CD56, together with the CD4 surface marker. To our knowledge, this is the first report on virus-specific CD4^+^ CTLs co-expressing CD56 antigen. Our results suggest a role for CD4^+^ CTL in B19 immunity. Such cells could function within both immune regulation and triggering of autoimmune phenomena such as systemic lupus erythematosus (SLE) or rheumatoid arthritis.

Human parvovirus B19 is a small DNA virus with a seroprevalence as high as 30–60% among adult population.^1^ Children usually get infected after entering school, yet 25% of the cases remain asymptomatic.^1^ Typical clinical manifestations of B19 infection are fifth disease and arthropathy. More severe clinical manifestations are also possible: acute anemia in patients with increased red cell turnover as well as neurological, myocardial and chronic infections.^1^ B19 infections have been suggested to set off or aggravate autoimmune ailments such as rheumatoid arthritis (RA) or systemic lupus erythematosus (SLE).^1, 2^ In addition to its natural target cells, the erythroid progenitor cells,^1, 3^ B19 DNA persists in various non-permissive tissues throughout life of the host.^4, 5^ Importantly, adenovirus co-infection may compensate for the failure of B19V DNA replication in non-permissive cells.^5^

B19 infection induces long-lasting antibody and cellular responses.^1, 3^ To date, both CD8^+^ T cells with cytotoxic potential^6, 7^ and CD4^+^ T cells with helper functions have been described^8, 9^ in B19-seropositive individuals.

CD4^+^ T cells may also have direct cytolytic potential (CD4^+^ CTLs).^10^ Such class II-restricted CTLs have significance in the pathogenesis of autoimmune diseases^11, 12^ and in the control of chronic viral infections such as EBV,^13^ CMV,^14^ HIV,^15^ as well as malignancies.^16, 17, 18^ Two major cell-killing mechanisms have been reported. One involves interaction of Th-cell surface antigen Fas ligand (Fas L) with the Fas antigen on the target cell surface.^19^ The other is the granule exocytotosis pathway, which employs perforin and serine proteases called granzymes.^20^ Both of these mechanisms culminate in activating caspases and inducing apoptosis in target cells.^10^ Granzymes, such as granzyme B (GrB), can also cleave other substrates besides caspases. This enzymatic activity may potentially contribute to autoimmunity by creating novel autoimmune epitopes from self-proteins.^21^ It can also mediate direct antiviral activity by cleaving essential viral proteins, as shown in adenovirus^22^ and herpes simplex virus models.^23^

Until now, no studies have explored whether in human parvovirus B19 infection CD4^+^ T cells with cytolytic potential are generated. This point is of special interest, since the clinical manifestations of B19 infection share some characteristics in common with conditions reported to induce cytotoxic CD4^+^ T-cell function: chronic infection and autoimmunity.

## Results

### GrB responses among the B19-seropositive and -seronegative subjects

B19, HBoV1 and *Candida albicans* antigens were all found to induce peripheral blood mononuclear cell (PBMC) to secrete GrB in 30 B19-seropositive and 22 B19-seronegative subjects (Table 1). HBoV1 and *Candida albicans* responses proved similar (*P*⩾0.258) among the B19-seropositive and -seronegative subjects, whereas B19-specific GrB responses were much stronger (*P*⩽0.0001) among the B19-seropositive than among the seronegative subjects (Table 1). All subjects had GrB responses ⩾384 pg ml^−1^ with the *Candida albicans* antigen. Next, the strength of HBoV1 and B19-specific GrB responses within the B19-seronegative and -seropositive subjects (Table 1) was compared using both antigens at the same (1.5 μg ml^−1^) concentration. Among the seronegative subjects, GrB responses proved significantly stronger with the HBoV1 than with the B19 antigen (*P*<0.04), whereas among the B19-seropositive subjects significantly stronger (*P*=0.005) responses were found with B19 than with HBoV1.

### Correlation analysis of GrB responses

**Figure 1:**
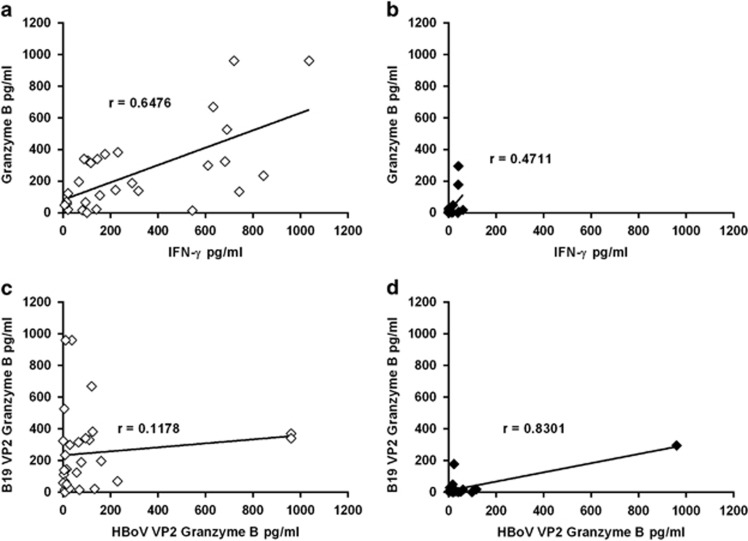
Correlation analysis. (**a**, **b**) Correlation between B19-specific IFN-γ and GrB responses among B19-seropositive (**a**) and -seronegative (**b**) individuals. (**c**, **d**) HBoV1 versus B19-specific GrB responses among the B19-seropositive (**c**) and -seronegative (**d**) subjects. Antigen concentrations were 1.5 μg ml^−1^. Spearman's correlation test was used.

First, correlations between the B19-specific interferon gamma (IFN-γ) and GrB responses were studied among the 30 B19-seropositive and 22 seronegative subjects. As shown in Figure 1a, a strong correlation (*P*<0.0001) was found between the IFN-γ and GrB in the B19-seropositive group, whereas the respective correlation was less significant (*P*=0.024) among the seronegative subjects (Figure 1b). No significant correlation (*P*=0.53) was found between HBoV1- and B19-specific GrB responses among the B19-seropositive subject (Figure 1c). Most of the B19-seronegative subjects showed virtually absent B19-specific GrB response (Figure 1c).

### Responder analysis of GrB responses

In all, 21 of the 30 B19-seropositive subjects were found to be ‘responders' having B19-specific GrB response ⩾50 pg ml^−1^ (Figure 1a). Two such responders were also detectable among the 22 B19-seronegative subjects (Figure 1b), *P* <0.0001. One of the two B19-seronegative ‘responder' showed weak concomitant HBoV1-specific response, whereas then other one showed vigorous HBoV1-specific response (Figure 1c).

### Perforin responses among the B19-seropositive and -seronegative subjects

**Figure 2:**
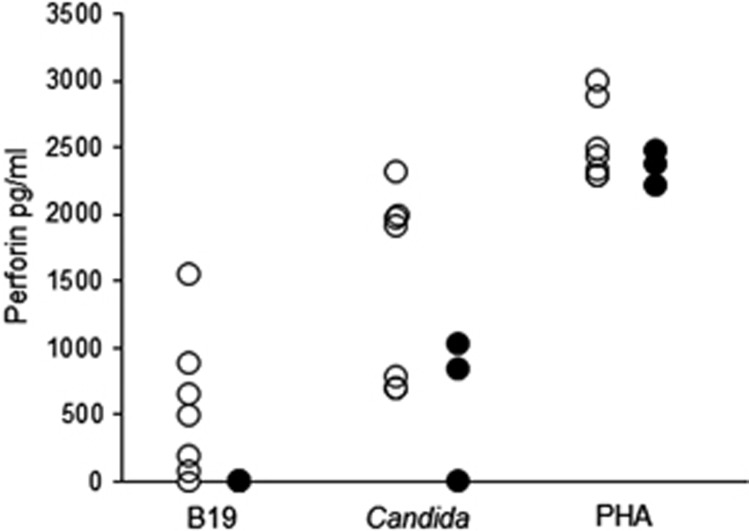
B19-specific perforin responses. Perforin responses in seven B19-seropositive (open circles) and three seronegative (closed circles) subjects with B19 VP2 VLPs, *Candida albicans* and PHA.

**Figure 3:**
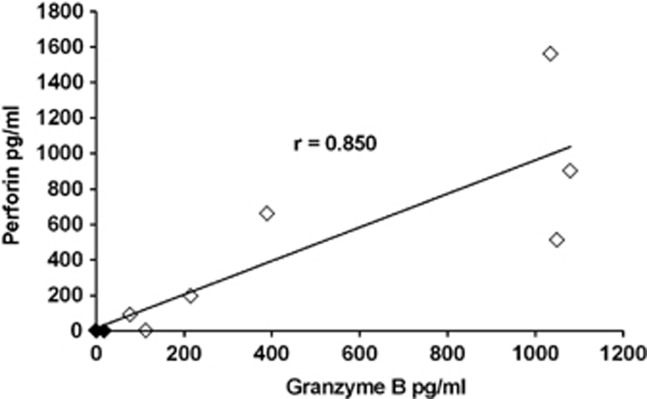
Correlation between B19-specific GrB and perforin responses. Correlation between B19-specific GrB and perforin responses among B19-seropositive (open quadrangles) and seronegative (closed quadrangles) individuals tested at antigen concentration 1.5 μg ml^−1^. Spearman's correlation test was used.

Perforin responses were studied in seven B19-seropositive and three seronegative subjects. B19-specific perforin responses were detectable only in B19-seropositive subjects, whereas PHA elicited strong responses in all and *Candida albicans* antigen in all but one subject (Figure 2). The strength of GrB responses had a significant correlation with the strength of perforin response (*P*=0.002) (Figure 3).

### Identification of the GrB- and perforin-secreting cells

**Figure 4:**
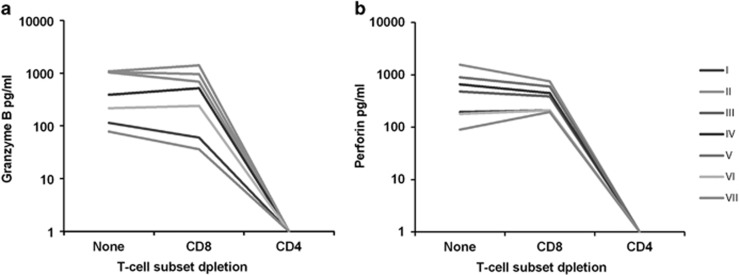
B19 VP2-specific cytolytic CD4^+^ T-cell responses after T-cell subset depletion. PBMC of seven B19-seropositive (I to VII) individuals were depleted of either CD4^+^ or CD8^+^ T cells and subjected to stimulation with B19 VP2-VLPs. T-cell responses were assessed by GrB (**a**) and perforin (**b**) ELISA.

To identify the perforin- and GrB-secreting cell populations, the PBMC was depleted of either CD4^+^ or CD8^+^ T cells by using monoclonal antibodies (MAbs) attached to magnetic beads. Secretion of both GrB (Figure 4a) and perforin (Figure 4b) was readily detectable after depletion of CD8^+^ T cells, whereas the removal of CD4^+^ T cells abrogated these responses among all the subjects (Figures 4a and b). GrB responses were also detectable after removal of CD8^+^, NK and B cells (supplementary Figure 1).

### HLA restriction of perforin- and GrB-secreting cells

**Figure 5:**
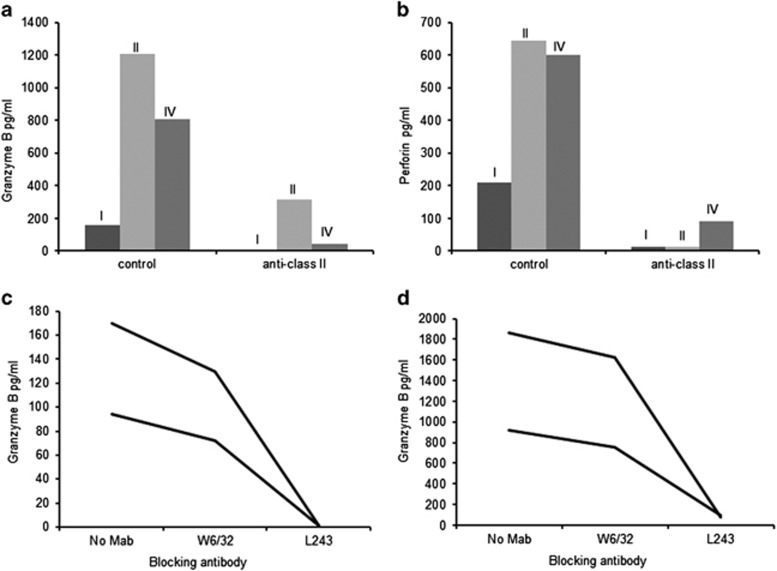
Effect of HLA-specific MAbs on B19 VP2-specific cytolytic CD4^+^ T-cell responses. (**a**, **b**) PBMC of three B19-seropositive individuals were blocked with HLA class II-specific MAbs and stimulated with B19-VP2 for GrB (**a**) and perforin (**b**) assessment. (**c**, **d**) PBMC of two B19-seropositive individuals with moderate (**c**) or strong (**d**) GrB responses were blocked with HLA class I-specific MAb W6/32 or with HLA DR-specific antibody L243 (MAbs) and stimulated with B19-VP2 for GrB assessment.

HLA class restriction of the perforin- and GrB-secreting cells was studied with a class II-specific MAb (which blocks antigen presentation) and with an isotype-matched control MAb among three B19-seropositive subjects. The perforin (Figure 5b) and GrB (Figure 5a) responses were readily detectable with the isotype control MAb, yet strongly reduced with the HLA class II-specific MAb (Figures 5a and b). Next, GrB responses were explored with respect to the effect blocking of antigen presentation via HLA class I or HLA-DR (HLA class II subclass). Two subjects with moderate (Figure 5c) and strong GrB responses (Figure 5d) were studied. The patterns of inhibition proved almost identical in these two groups: class I inhibition had a minimal effect, whereas inhibition of presentation via HLA-DR virtually abrogated GrB responses (Figures 5c and d).

### Direct cytotoxicity of B19-specific CD4^+^ T cells

**Figure 6:**
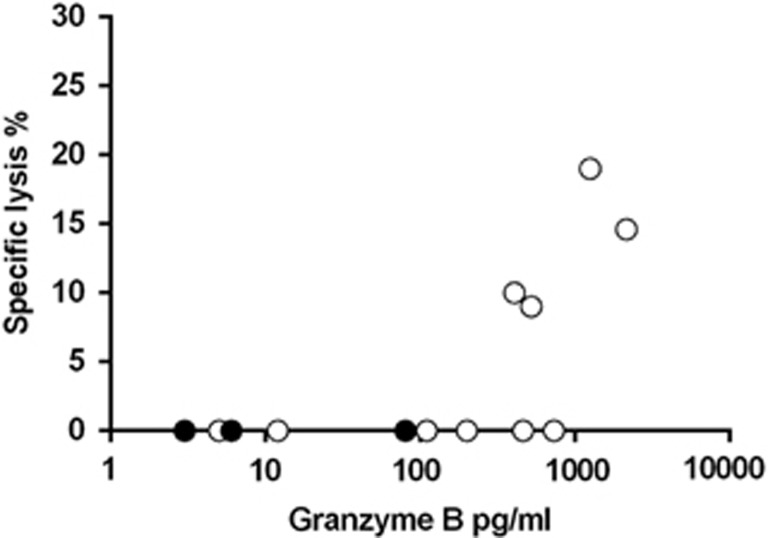
Direct cytotoxicity versus GrB secretion among seven B19-seropositive subjects. Cells were incubated for 5 days. Cytotoxicity and GrB responses among seropositive subjects (open circles) and seronegative subjects (closed circles) were determined simultaneously. B19 VLP was used at a concentration of 1.5 μg ml^−1^.

Next, B19-specific CD4^+^T cells were explored for direct cytotoxicity. Ten B19-seropositive and three seronegative subjects (age range 30–58 years) were studied. While no cytotoxicity was observed after 3-day culture (data not shown), 5-day culture gave direct cytotoxic responses, especially in subjects with strongest concomitant GrB responses (Figure 6).

### Intracytoplasmic staining of B19-specific PBMC

**Figure 7:**
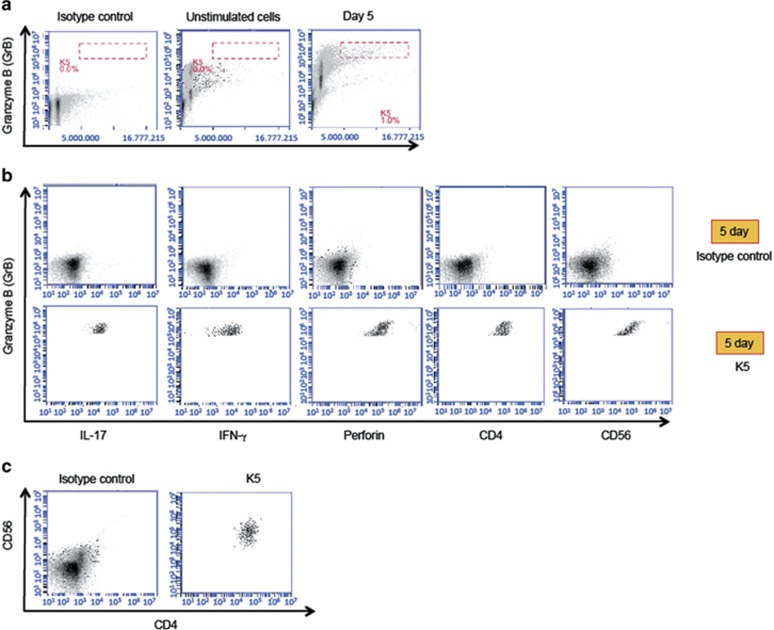
Expression of surface markers and intracellular proteins in B19-specific PBMC. (**a**) PBMC from a subject ‘K' were incubated for 5 days with B19-VLPs (left and right panels) or with media alone (middle panel). Then B19-specific cells with highest intracellular GrB signal and forward scatter were gated (gate K5, right panel) for further analysis. Such cells were absent in unstimulated cells stained with GrB-antibody (middle panel) or B19 stimulated cells stained with GrB-isotype control antibody (left panel). (**b**) IL-17, IFN-γ, perforin, CD4 and CD56 expression signals in K5-gated cells are shown. (**c**) Left panel: total ungated PBMC stimulated with B19 VLPs and stained with isotype controls for CD4 and CD56 antigens. Right panel: co-expression of CD4 and CD56 antigens in B19-specific PBMC in gate K5.

Finally, we carried out intracellular cytokine staining (ICS) experiments to characterize further the nature of B19-specific CD4^+^ T cells. Three subjects (age range 56–58 years) known to have strong B19-specific GrB responses were studied, and all showed representative results. These subjects have been B19-seropositive for years or decades. Data obtained with subject ‘K' are shown in Figure 7. Cells activated by B19 VLPs were identified by high forward scatter combined with a strong intracellular GrB signal (Figure 7a, right panel). These cells were gated for further analysis. Such PBMC populations were absent among PBMC cultured without B19 VLPs (Figure 7a, middle panel) or among PBMC cultured with B19 VLPs and stained with isotype control (Figure 7a, left panel). B19-activated cells could be identified already after 3 days of culture (data not shown), and after 5 days they had been expanded further (Figure 7a). As expected by previous results, GrB was secreted by CD4^+^ T cells, and co-expression of GrB and perforin, as well as GrB and IFN-γ was detected (Figure 7b).

Unexpectedly, B19-specific CD4^+^ T cells also showed (1) a bright fluorescence after interleukin 17 (IL-17) staining (Figure 7b), and (2) a co-expression of CD4 and CD56 surface markers (Figure 7c). Similar data were obtained with subjects ‘L' and ‘H' (Supplementary Figure 2).

## Discussion

Th cells serve a traditional function as essential regulators of B cells and CD8^+^ T cells. Recently, data proving that a direct cytolytic role can be ascribed to CD4^+^ T cells have emerged both in murine^24, 25^ and in human^10, 14, 15^ models. The latter suggest that these cytotoxic CD4^+^ cells have a part in controlling chronic viral infections such as EBV, CMV and HIV.^13, 14, 15^

Since B19 has been observed to establish a chronic infection, and to be linked with autoimmunity, it was of interest to explore whether such CD4^+^ CTLs could also emerge after B19 infection. We studied 30 asymptomatic B19-seropositive and 22 seronegative subjects by using B19 VP2 VLPs as antigens. During natural B19V infection, similar empty capsids are formed along with infectious virions.^5^ HBoV1 VLPs and heat-inactivated *Candida albicans* were used as controls. The present study focused primarily on GrB, not only for its critical role in cytolysis, but also because of emerging data on its function as an inducer of autoimmunity.^21^ However, to mediate cytolysis GrB needs delivery into target cells by perforin.^20^ Therefore, it was also important to study B19-specific perforin responses. We (1) found a strong correlation between B19-induced perforin and GrB secretion, (2) showed by T-cell subset depletion and HLA-class II blocking assays that the responses were largely confined to CD4^+^ T cells, and (3) confirmed with ICS that GrB and perforin are produced in the same B19-specific CD4^+^ T cells. However, in some subjects also HLA-class I blocking and removal of CD8^+^ T cells slightly reduced B19-specific GrB secretion. This suggests that B19 VP2-VLPs might also have a minimal potential to stimulate CD8^+^ T cells in some individuals, by the process of cross priming.^26, 27^

The B19-specific GrB responses proved much stronger in the seropositive than in the seronegative subjects, indicating that GrB was secreted by cells, which establish memory. The GrB responses proved significantly stronger with B19 VLPs than with HBoV1 VLPs among the seropositive subjects, whereas a reverse pattern was observed among the B19-seronegative subjects. Since B19 viral DNA establishes a lifelong persistence,^28^ the B19-specific GrB responses observed suggest that CD4^+^ T cells secreting GrB may contribute to the surveillance of B19 by guarding against reactivation in cases where viral helper function is provided by other viruses.^29^ By contrast, the other human parvovirus HBoV1 does not establish comparable long-term persistence;^30^ consistently, the GrB responses to this latter antigen in the present study proved low.

Exploring direct cytotoxicity by a lactate dehydrogenase (LDH) release assay revealed B19-specific CD4^+^ T cells with direct cytotoxicity among subjects with strongest GrB responses; also some of those with moderate GrB responses showed cytotoxicity. In our experimental setup, the target (or antigen-presenting) cells were CD4- and CD8-depleted PBMC, that is, monocytes. Direct cytotoxicity might be more readily detectable by using B19-specific B cells as targets, since regulating B-cell immunity appears to be one of the key roles of B19-specific CD4^+^ CTLs.

Among the B19-seropositive subjects, there was a clear correlation between B19-specific IFN-γ and GrB responses, while no correlation was found between the HBoV1- and B19-specific GrB responses. ICS expreriments confirmed the co-expression of GrB and IFN-γ in B19-specific CD4^+^ T cells. Thus, also in the B19 model, consistent with previous reports,^21^ GrB appeared to be secreted by Th1-like cells, and the vigor of the GrB response was associated with that of antigen-induced Th-cell activation.^31^ This was further supported by the co-expression of CD4 and CD56 or neural cell adhesion molecule-1 found among the B19-specific CD4^+^ T cells. In previous studies, CD4^+^ T, CD8^+^ T and γδ T cells co-expressing CD56 antigen have shown enhanced cytotoxicity.^32, 33, 34, 35, 36^ On the other hand, CD4^+^ T cells co-expressing CD56 (NKT-like cells) have proved to be important mediators in autoimmune diseases such as multiple sclerosis,^37^ Behçet's disease^38^ and type-1 diabetes.^34^

At present, the literature focusing on co-expression of CD4 and CD56 antigens among pathogen-specific CD4^+^ T cells is almost lacking. Taddesse-Heath *et al.*^39^ used immuno-histochemical methods and found an infiltrate of CD4^+^CD56^+^ T cells in a nasopharyngeal mass induced by herpes simplex virus. They presumed that these CD4^+^CD56^+^ T cells represented florid antiviral immune response. To our knowledge, the present report is the first time to describe a co-expression of CD4 and CD56 antigens in definite virus-specific cytolytic CD4^+^ T cells.

The B19-specific CD4^+^ T cells showed also an intracellular IL-17 signal. IL-17 is a pro-inflammatory cytokine with important antibacterial and antifungal effects.^40^ Direct antiviral effects of IL-17 have been detected in vaccinia virus^41^ and hepatitis B virus models.^42^ On the other hand, inadequately regulated IL-17 responses have been linked to various autoimmune phenomena such as multiple sclerosis,^43, 44^ RA^45, 46^ and inflammatory bowel diseases.^47, 48^ Expression of intracellular IL-17 is also associated with T-cell activation. By using influenza-A model, Xie *et al.*^49^ showed that all activated human T cells co-expressed IL-17 and GrB. We believe that in our B19 model the co-expression of intracellular IL-17 and GrB is also linked to the strong activation of B19-specific CD4^+^ T cells.

Various autoimmune phenomena including the induction of autoantibodies and autoimmune diseases such as RA and SLE have been linked to B19 infection.^2, 50^ However, the pathogenetic mechanisms of B19-induced autoimmune diseases are not fully understood. Several mechanisms have been proposed: activation of the IL-6 and TNFα promoters by B19 NS1 protein during persistent infection,^51, 52^ molecular mimicry between a B19 VP2 epitope and autoantigens such as collagen II^53^ and the phospholipase activity of B19 VP1 unique domain with subsequent activation of synoviocytes^54^ and induction of anti-phospholipid antibodies.^55^ Recently, B19 NS1 was shown to induce apoptotic bodies containing self-antigens potentially associated with autoimmunity.^56^

We believe that GrB-secreting CD4^+^ T cells may be important players in the autoimmune processes triggered by B19 infection. First, CD4+ T cells with cytolytic potential have been described in patients with RA^11^ and SLE.^12^ Second, GrB has been shown to cleave autoantigens and create unique fragments recognized by autoantibodies.^57, 58^ Third, besides cleaving intracellular substrates, GrB can function extracellularly^59^ and mediate tissue destruction by degrading substrates such as cartilage proteoglycan,^60, 61^ and proteins involved in extracellular structure and function: vitronectin, fibronectin and laminin.^62^ Finally, as T-cell receptors appear to be extremely cross-reactive,^63, 64, 65, 66^ it may be possible that the GrB-secreting CD4^+^ T cells induced originally by B19 are later activated by other pathogens—without any evidence of B19 being reactivated.

In conclusion, our study is the first one to show B19 antigen-specific CD4^+^ T cells with cytolytic potential. These cells may have a part in B19 virus elimination and control. The pathogenetic role of these B19-specific CD4^+^ T cells secreting GrB (and possibly IL-17) in autoimmune diseases such as RA and SLE warrants further study.

## Methods

### Study groups

Altogether 52 voluntary, asymptomatic subjects (age range 23–58 years) were enrolled, of whom 30 proved seropositive and 22 seronegative for B19, and all were seropositive for human bocavirus (HBoV1). In addition, three B19-seronegative subjects (females aged 21–42 years) participated in the perforin experiments.

### Ethics statement

Ethical approval was received from the ethics committee of the University of Helsinki. Informed written consent was obtained from every subject.

### Antibody assays

IgG for B19 and HBoV1 in plasma were measured by in-house enzyme immunoassays employing as antigen virus-like particles.^8, 67^

### Antigens

The B19 and HBoV1 VP2 VLPs were expressed, purified and sterilized as described.^8, 9, 67^ The antigens were further characterized by SDS-PAGE with silver staining (SilverXpress, Invitrogen) and immunoblotting with B19- and HBoV1-seropositive human sera.^8, 9, 67^ In-house prepared and heat inactivated *Candida albicans* was used as a second control antigen. Endotoxin content in the antigen preparations as measured by the Limulus amebocyte lysate assay (QCL-1000; Cambrex Biosciences, Walkersville, MD, USA) was found to be less than 2 EU mg^−1^ for both viral antigens.

### Isolation of PBMC

Blood was drawn to mononuclear cell separation tubes (Vacutainer CPT, Becton Dickinson, Franklin Lakes, NJ, USA) containing 0.45 ml sodium citrate. The tubes were centrifuged at 1500 *g* for 30 min and washed two times with PBS. PBMC were separated within 2 h of blood sampling followed by counting.

### Lymphocyte culture

Lymphocyte culture was conducted as described previously.^68^ Briefly, isolated PBMC were resuspended in the RPMI-1640 medium (Sigma) containing 20 mM HEPES, 2 mM L-glutamine, streptomycin (100 μg ml^−1^), penicillin (100 U ml^−1^), 50 μM 2-mercaptoethanol and 10% human AB serum (Cambrex Biosciences) and were cultured with the antigens. B19 VP2 VLPs were used at 1.50 and 0.5 μg ml^−1^, and the HBoV1 VLP and *Candida albicans* control antigens at 1.50 and 2.5 μg ml^−1^, respectively.

### IFN-γ, perforin and GrB detection

The PBMC culture supernatants were harvested for perforin, GrB and IFN-γ after 3 days and stored at −20 °C. In the first phase, cytokine responses from the 52 subjects were analyzed by GrB platinum ELISA^69^ (eBiosciences, San Diego, CA, USA) and IFN-γ (Pharmingen, San Diego, CA, USA) kits, according to the manufacturers' instructions. Later, perforin and GrB responses were compared by using MABTECH ELISA kits for perforin and GrB (MABTECH AB, Nacka Strand, Sweden). Background cytokine production was subtracted from total to yield antigen-specific cytokine production.

### Depletion of CD4^+^ or CD8^+^ cells

PBMC were depleted of CD4^+^ or CD8^+^ T cells by using magnetic beads coated with CD4- or CD8-specific MAbs (Invitrogen Dynal AS, Oslo, Norway), according to the manufacturer's instructions. Then, 200 000 pure CD4^+^- or CD8^+^- depleted cells were cultured with the antigens as described.^68^ The purity of cell populations was analyzed by BD Accuri C6 flow cytometer (Becton Dickson, San Jose, CA, USA) at Biomedicum Flow Cytometry Core Facility, University of Helsinki. The total and the CD4- and CD8-depleted PBMC populations were washed twice with PBS and incubated for 30 min at +4 °C with MultiMix triple-color cocktail of FITC, RPE and APC labelled MAbs specific for CD8, CD4 and CD3, respectively (DakoCytomation, Glostrup, Denmark). Isotype-control antibodies (DakoCytomation) were used in parallel. A depletion efficiency of >95% was verified by flow cytometry for both CD4 and CD8 depletions (data not shown).

### Antibody blocking assays

Class restriction of the T-cell responses was first studied by HLA class II-specific MAbs (HLA-DR, DP, DQ) (IgG2a, clone Tu39; BD Pharmingen, San Diego, CA, USA), or isotype control MAbs (IgG2a, clone G155-178; BD Pharmingen). These antibodies were used at 10 μg ml^−1^. Class restriction was studied further by comparing the effect of HLA-DR-specific MAbs (IgG2a, clone L243, Abcam, Cambridge, UK) and HLA class I-specific MAbs (IgG2a, clone W6/32, Abcam). These antibodies were used at 1.25 μg ml^−1^.

### Cytotoxicity assay

CD4^+^ T cell-mediated direct cytotoxicity was studied by using a LDH release assay (Pierce LDH cytotoxicity assay kit, Thermo Fisher Scientific Inc, IL, USA). First, PBMC were depleted of CD4^+^ T cells and then of CD8^+^ T cells by using magnetic beads coated with CD4- and CD8-specific MAbs as described above. The CD4- and CD8-depleted PBMC were used as target cells. Positively isolated CD4^+^ cells were detached from beads using DetachaBeads (Invitrogen Dynal AS) and used as effectors. Cytotoxicity was determined as instructed. Briefly, 50 000 target cells were cultured either alone (to determine the spontaneous and maximum LDH release) or with 100 000 pure CD4^+^ T cells and B19 VLPs at 1.5 μg ml^−1^ (to determine experimental lysis). In all, 100 000 CD4^+^ T cells were also cultured alone to determine their spontaneous LDH release. Maximum lysis was determined by lysing target cells with lysis buffer. After 3 or 5 days, LDH release was measured with spectrophotometer from supernatants. Cytotoxicity was determined as follows:


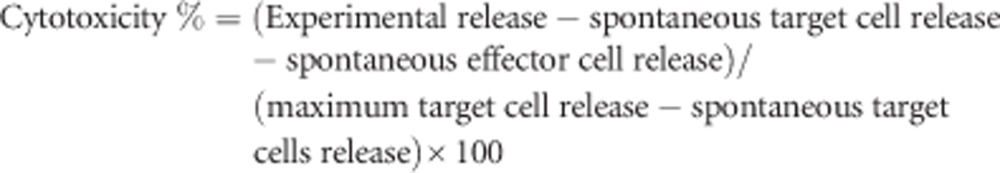


Simultaneously, the GrB responses were determined by culturing 100 000 effectors and 50 000 targets with B19 VLPs at 1.5 μg ml^−1^ or tissue culture media (background response). Next, background response was subtracted from antigen-induced response.

### ICS experiments

PBMC were incubated with B19 VLPs or with tissue culture media alone as described above. ICS was done after 3- or 5-day culture. Briefly, Fc receptors of PBMC were first blocked with highly purified and concentrated human immunoglobulin G (Gammagard, Baxter, Westlake Village, CA, USA). Then 8 × 10^5^ PBMC were stained for surface antigens with CD4- and CD56-specific MAbs for 30 min at 4 °C and washed three times with staining buffer (10% FBS in PBS). Next, cells were fixed and permeabilized with BD Cytofix/Cytoperm solution as instructed by the manufacturer and stained for intracellular GrB, perforin, IFN-γ and IL-17 with respective specific MAbs for 30 min at 4 °C in the dark, washed three times in Perm/wash solution and resuspended in staining buffer. The samples were analyzed on a BD Accuri C6 Flow Cytometer with the software provided by the manufacturer. PBMC activated by B19 VLPs were identified by using a method developed by Böhmer et al.^70^ In this method, antigen-activated cells are identified by detecting simultaneous high forward scatter and strong activation signal; in our setup the signal was intracellular GrB. PBMC with highest forward scatter and intracellular GrB signal were gated for further analysis. Such PBMC populations were absent among PBMC cultured without B19 VLPs.

The following antibodies from BD Biosciences were used in the volumes suggested by the manufacturer: CD4 FITC-IgG1, CD56 PECy5-IgG1, GrB PE-IgG1, Perforin AF647-IgG2b, IL-17 AF647-IgG1 and IFN-γ PECy7-IgG1 (final concentration 50 ng ml^−1^). Isotype and fluorochrome-matched negative control MAb were used as controls (Abcam).

### Statistical methods

Responses between B19-seropositive and seronegative subjects were compared with Mann–Whitney *U* test. Paired HBoV1- and B19-specific responses were analyzed with Wilcoxon Signed Rank Test, and the correlation of GrB responses with IFN-γ and perforin responses was studied with Spearman's correlation. The presence of B19-specific ‘responders' (having a B19-specific GrB response of ⩾50 pg ml^−1^) were compared by Fisher's Exact Test. *P*-values⩽0.05 were considered as significant.

## Figures and Tables

**Table 1:** Comparison of B19-specific Granzyme B (GrB) responses among the 30 B19-seropositive and 22 seronegative subjects

*B19 serostatus*	*Candida albicans*	*HBoV*	*B19 0.5 μg ml*^−1^	*B19 1.5 μg ml*^−1^
Positive	832.0 (444–958)	111.3 (0–960)	127.8 (0–960)	247.0 (0–960)
Negative	805.8 (384–954)	71.3 (0–960)	22.6 (0–237)	29.9 (0–295)
*P*^a^	0.258	0.517	<0.0001	<0.0001

Mean and range (in bracket) are shown.

a Mann–Whitney *U* test.
