# Supplementary material for: Granzyme B mediated function of Parvovirus B19-specific CD4+ T cells
Source: Clin Transl Immunology. 2015 Jul 3;4(7):e39–. doi: 10.1038/cti.2015.13 (PMC4524951; doi:10.1038/cti.2015.13)
Supplement: Supplementary Information [file cti201513x1.doc]

**Supplementary Figure 1** VP2–specific GrB- responses after depletion of CD8^+^, CD19^+^ (B-cells) and NKp46^+^ (NK-cells)**.** PBMC of three B19 seropositive (I to III)) individuals were depleted of CD8^+^ T cells, B-cells and NK-cells with Dynabeads®, and subjected to stimulation with B19 VP2-VLPs in the presence of isotype-control antibody or HLA-class II blocking MAb . T cell responses were assessed by GrB ELISA.

**Supplementary Figure 2** Expression of surface markers and intracellular proteins in B19-specific PBMC from subjects "H" (A) and "L" (B). (A) I. PBMC from a subject "H" were incubated for five days with B19-VLPs (left and right panels) or with media alone (middle panel). Then B19-specific cells with highest intracellular GrB signal and forward scatter were gated (gate H5, right panel) for further analysis. Such cells were absent in unstimulated cells stained with GrB-antibody (middle panel) or B19 stimulated cells stained with GrB-isotype control antibody (left panel).

(A) II. IL-17, IFN-γ, perforin, CD4 and CD56 expression signals in H5-gated cells are shown.

(A) III. (C) Left panel: total ungated PBMC stimulated with B19 VLPs and stained with isotype controls for CD4 and CD56 antigens. Right panel: co-expression of CD4 and CD56 antigens in B19-specific PBMC in gate H5.

Similar results were obtained with subject "L" as shown in (B) I, II and III.
