# Supplementary figures and images for: Granzyme B mediated function of Parvovirus B19-specific CD4+ T cells
Source: Clin Transl Immunology. 2015 Jul 3;4(7):e39–. doi: 10.1038/cti.2015.13 (PMC4524951; doi:10.1038/cti.2015.13)

**Supplementary Fig. 1**

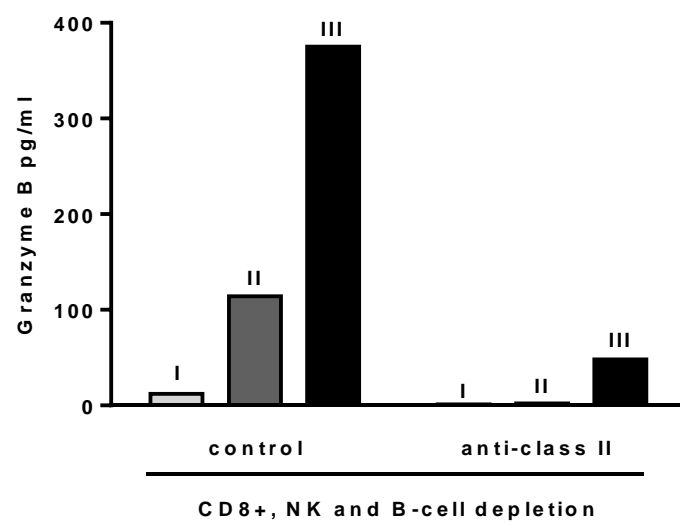

Supplement: Supplementary Figure 1 [file cti201513x2.pdf]

Supplementary Fig. 2

(A)

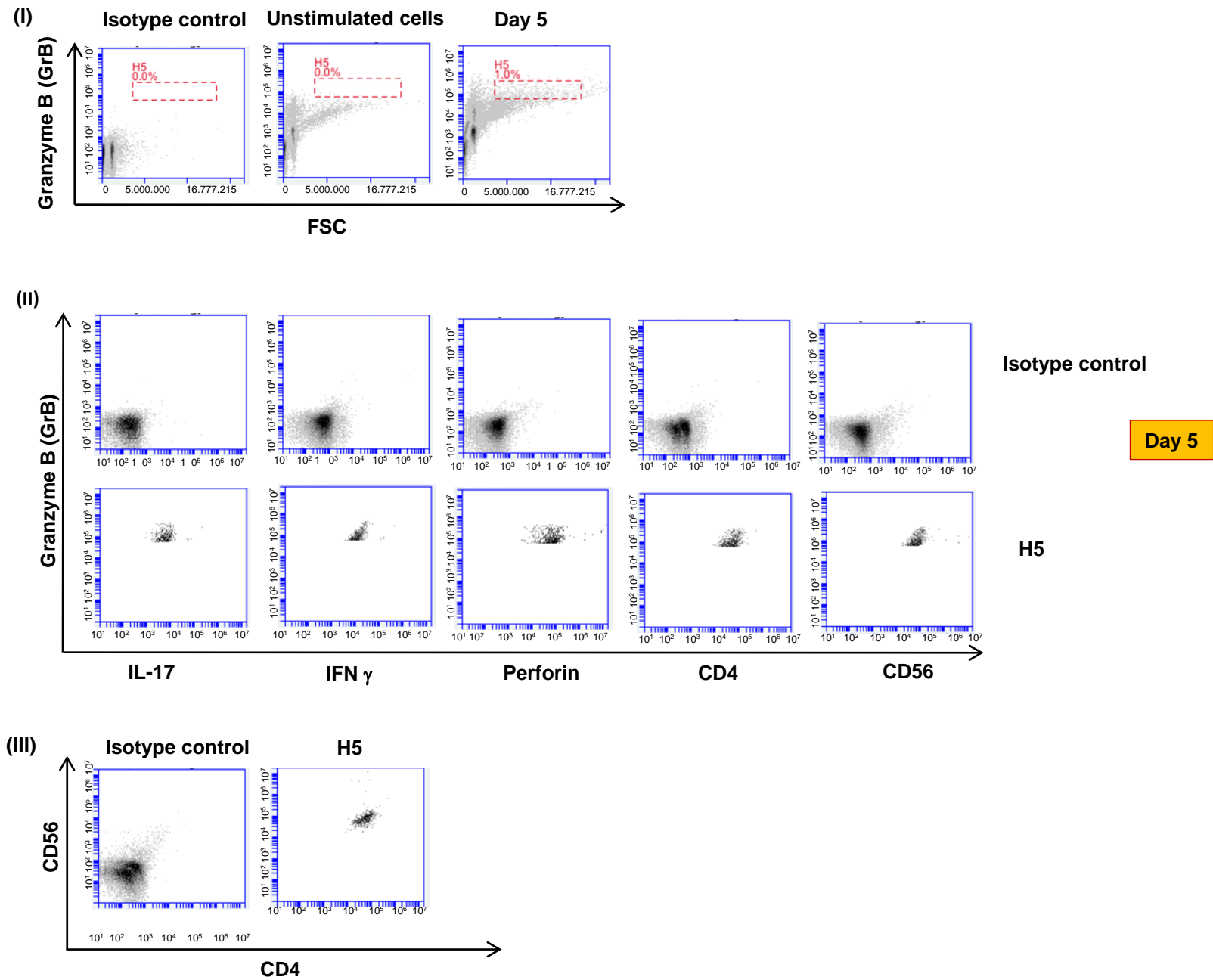

(B)

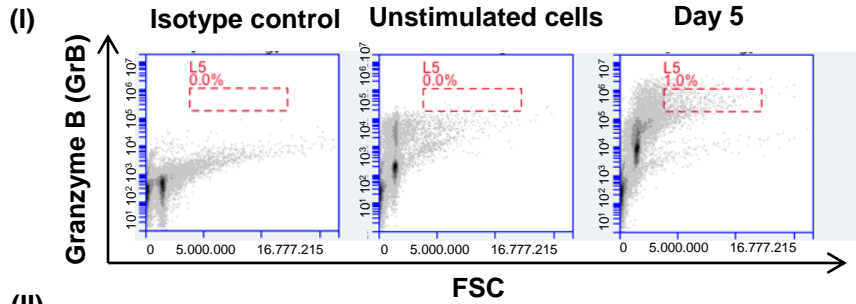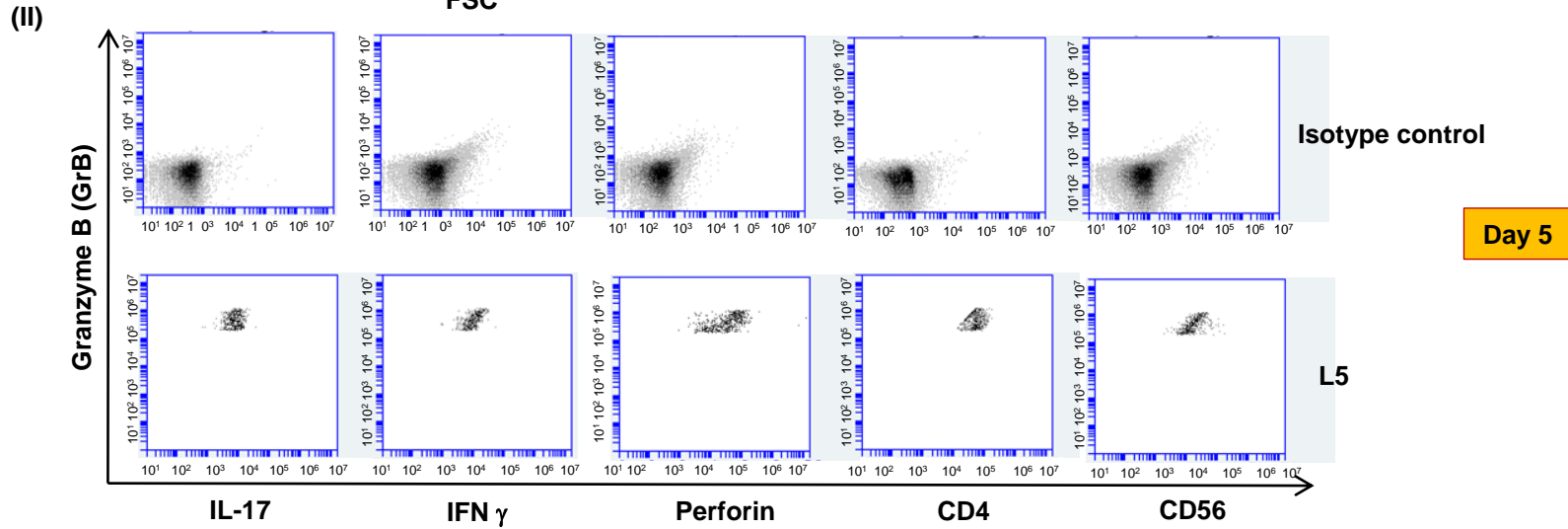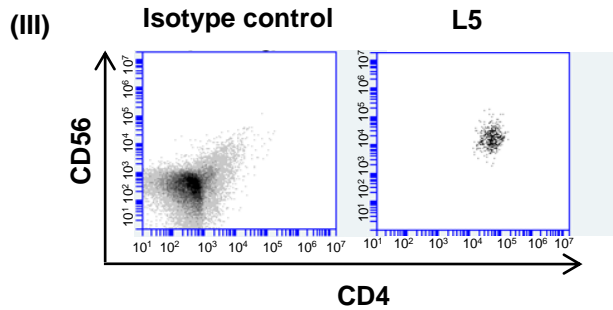

Supplement: Supplementary Figure 2 [file cti201513x3.pdf]
